# MR-guided HDR prostate brachytherapy with teleoperated steerable needles

**DOI:** 10.1007/s11701-023-01676-x

**Published:** 2023-07-22

**Authors:** M. de Vries, M. Wijntjes, J. Sikorski, P. Moreira, N. J. van de Berg, J. J. van den Dobbelsteen, S. Misra

**Affiliations:** 1 Department of Biomechanical Engineering, Delft University of Technology, Delft, The Netherlands; 2 Department of Biomechanical Engineering, University of Twente, Enschede, The Netherlands; 3 Department of Radiology, Brigham and Women’s Hospital, Harvard Medical School, Boston, USA; 4 Department of Gynaecological Oncology, Erasmus MC Cancer Institute, Rotterdam, The Netherlands; 5 Department of Biomedical Engineering, University of Groningen and University Medical Center Groningen, Groningen, The Netherlands

**Keywords:** Prostate, Brachytherapy, Steerable needle, MR-guided, Teleoperation, MIRIAM, daVinci Surgical System

## Abstract

Conformity of tumour volumes and dose plans in prostate brachytherapy (BT) can be constrained by unwanted needle deflections, needle access restrictions and visualisation limitations. This work validates the feasibility of teleoperated robotic control of an active steerable needle using magnetic resonance (MR) for guidance. With this system, perturbations can be counteracted and critical structures can be circumvented to access currently inaccessible areas. The system comprises of (1) a novel steerable needle, (2) the minimally invasive robotics in an MR environment (MIRIAM) system, and (3) the daVinci Research Kit (dVRK). MR scans provide visual feedback to the operator controlling the dVRK. Needle steering is performed along curved trajectories to avoid the urethra towards targets (representing tumour tissue) in a prostate phantom with a targeting error of 1.2 ± 1.0 mm. This work shows the potential clinical applicability of active needle steering for prostate BT with a teleoperated robotic system in an MR environment.

**Supplementary Information:**

The online version contains supplementary material available at 10.1007/s11701-023-01676-x.

## Introduction

Prostate cancer is the second most common cause of cancer related deaths among men [1]. High-dose-rate (HDR) brachytherapy (BT) is a form of internal radiotherapy with excellent clinical outcomes for localized prostate cancer [2]. This technique ensures high radiation dose to the target volume while sparing surrounding healthy tissue. For this purpose, radioactive sources are temporarily placed in the target volume using rigid needles and transrectal ultrasound (TRUS) visualisation for guidance [3]. A crucial factor of obtaining conformal dose coverage is high targeting accuracy of the inserted needles [4]; inaccurate needle positioning can lead to misplacement, and consequently, radiation hot- or cold-spots. Unfortunately, TRUS imaging presents limited visual feedback of the internal structures and lesions [5], while needle tip visualisation can be difficult [4, 6, 7]. To complement TRUS visualisation, magnetic resonance imaging (MR, MRI) has been incorporated in BT protocols for treatment planning. This technique provides anatomical data and functional information of the prostate gland including potential lesions [8]. The disadvantage of MRI-TRUS visualisations is that images are collected at different moments and typically in different rooms, resulting in patient motions and introducing inaccuracies[9, 10].

MR-guided prostate BT does not require patient repositioning, and has demonstrated its feasibility in the last decades [11]. This approach posed new challenges such as the use of non-magnetic instrumentation only, and limited workspace within the MR scanner. Accordingly, MR-compatible robotic systems have been developed to perform prostate BT under MR guidance inside the MR scanner [11–13]. Previously, we have developed the MR-compatible “Minimally Invasive Robotics in an MR environment” (MIRIAM) system [14], with a 5 degree of freedom (DoF) parallel robot and 4 DoF driver for insertion and steering of a bevel-tip biopsy needle under MR-guidance.

Most robotic BT systems are focused on low-dose-rate (LDR) [11, 15, 16], and some on HDR solutions [17, 18]. The systems typically provide needle guide conditions for straight path insertions. To reduce needle misplacements, several developments have aimed to improve the needle placement accuracy, or enhance the accessibility of a tumour located ventrally to the urethra or behind the pubic arch, or avoid the penile bulb or neurovascular bundles [10, 19–22]. Earlier studies showed that increased accessibility of the prostate could improve dosimetric outcomes [22–25]. To reduce placement errors, Lagerburg et al*.* developed a robot that performed needle tapping during insertion [26], while other robots performed needle rotation to reduce insertion forces and improve targeting accuracy [27, 28]. Steerable needles have been developed to follow curved trajectories and counteract unwanted deflections by actively manipulating the distal tip during the insertion which is manually performed [29–31]. A review of the literature by Li et al. presents an exhaustive overview of different systems developed [32]. In the work of Kohn et al*.*, a tendon-driven steerable needle system for prostate HDR BT is presented [33]. For needles with compliant parts, local rigidity needs to be carefully attuned, to maintain steerability, mitigate hazards (snapping, buckling), and uphold needle state predictability. It was shown in the study of de Vries et al*.* that active HDR BT needles can be developed without significantly reducing the axial and flexural rigidity, thus ensuring controllability of the needle trajectory and accurate targeting in various inhomogeneous tissues [29]. Needle steering was possible regardless of the initial insertion depth.

The aim of this work is to validate the feasibility of teleoperated and MR-guided robotic control of an actively steerable HDR BT needle. It, therefore, combines previously developed components and systems, comprising of the MIRIAM system and the da Vinci Research Kit (dVRK), for MR-guided HDR prostate BT using steerable needles. The system combines high precision of piezoelectric actuation and real-time control of the steerable needle. The multiple degrees of freedom (DoFs) of the system enable control from outside the operating room over the orientation, position, and the degree of steering of the needle. The system architecture and workflow are outlined and teleoperation of the steerable needle is performed in an experiment with a developed prostate phantom in the MR scanner. The purpose of this work is to show the feasibility of the MR-compatible system and the successful workflow by inserting the steerable needle along a curved trajectory towards an obstructed target in the prostate tissue phantom under MR guidance. With this, we highlight the ability to perform teleoperated adaptive BT in the MR bore with increased accessibility to the prostate gland.

## Materials and methods

### System components

The teleoperated system integrates three subsystems: (1) the developed MR-compatible steerable needle, based on the design described by de Vries et al*.* [29], (2) the MIRIAM system designed by Moreira et al*.* [14] and (3) components from the daVinci Surgical System which is widespread available in hospitals used for robotic-assisted surgery [34, 35].

### Steerable needle

**Fig. 1:**
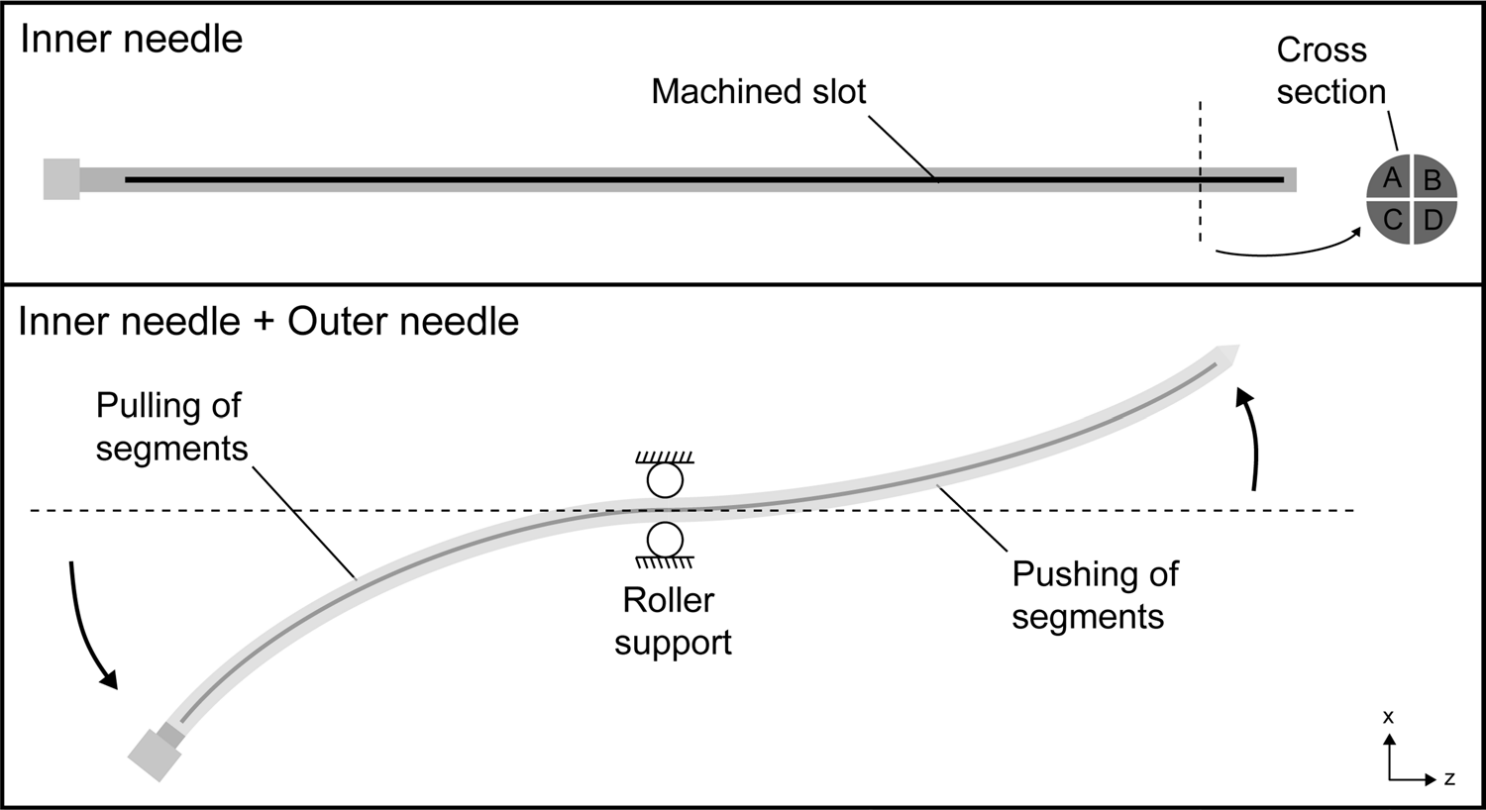
Needle steering mechanism. Bending the proximal end of the steerable needle introduces longitudinal movement of the four segments and steering of the distal end in the opposite direction. The needle guide is a roller support allowing for longitudinal movement of the steerable needle while constraining off-axis movement

The steerable needle comprises of a flexible outer needle with conical tip of polyoxymethylene (ProGuide sharp 6F needle, Elekta Instrument AB, Stockholm, Sweden) and a superelastic nitinol inner needle. The outer needle has a diameter of 2 mm and length of 240 mm. The inner needle is a 270 mm long single-piece rod with a diameter of 1.46 mm, machined into four segments by electric discharge machining along the longitudinal axis of the rod while keeping both ends of the rod connected. The four segments are shown in cross-section in Fig. 1. As a result, bending the proximal end of the steerable needle down results in a pulling force in upper sections A and B, and a pushing force in lower sections C and D, which are transferred through the segments of the inner needle. The outer needle was not altered. It is still the standard medical product. It prevents sideways movement of the segments which results in distal tip steering in the opposite direction while the needle guide functions as pivot point. After placement, the steerable inner needle is retracted and the outer needle is connected to the BT afterloader for the introduction of the radioactive source.

### MIRIAM system

**Fig. 2:**
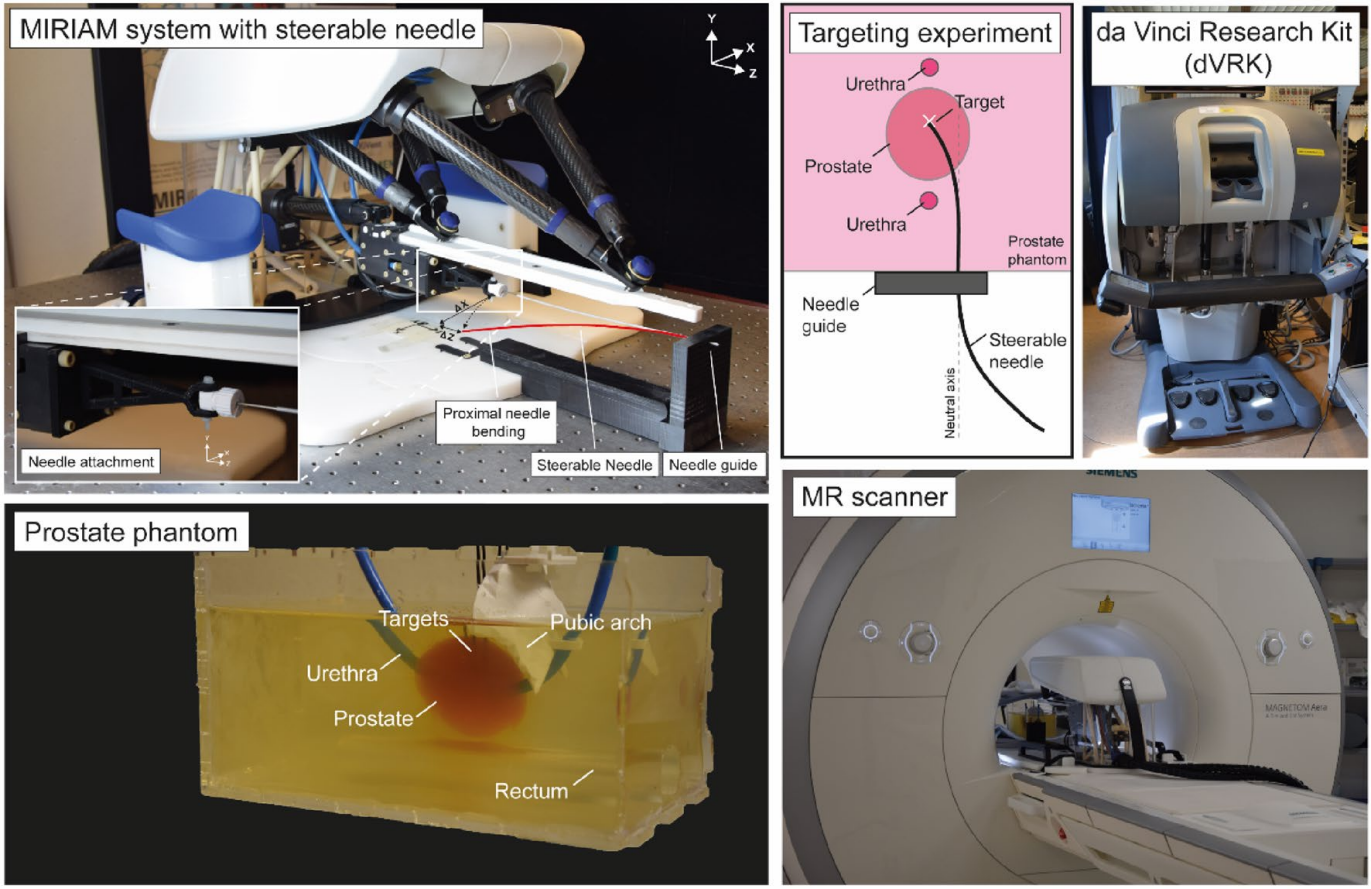
System components and schematic of the targeting experiment. The MIRIAM system holds the steerable needle and is placed in the 1.5 T MR scanner. The da Vinci Research Kit (dVRK) is placed outside the MR scanner room and the operator controls the MTM of the dVRK to perform the targeting experiment in which the needle is steered towards a pre-defined target in the prostate phantom. The system is connected via Ethernet using the local area network

The MIRIAM system for prostate biopsies consists of nonmagnetic components, and the low-level controller and motor drivers of the MIRIAM system are located in a controller cabinet outside of the MR scanner room to minimize electromagnetic interference in the MR scanner and is connected using 10-m-long shielded cables going through the waveguide [14]. The system is adapted for the purpose of BT omitting the functionality to collect tissue samples for diagnostics via the needle firing system. The adapted MIRIAM system facilitates the attachment and alignment of the steerable needle using a hinge joint and needle guide for guidance as shown in Fig. 2. The needle base can be moved using a 5-DoF parallel robot actuated by HR2 and HR8 piezo-electric motors (Nanomotion, Yoqneam, Israel). The base structure is made from ceramic rods and the needle base is positioned using five extendable carbon fibre-reinforced rods.

### da Vinci Research Kit

The MIRIAM system is controlled by the dVRK containing components from da Vinci Surgical Systems (Intuitive Foundation, Sunnyvale, CA, USA) and used for minimally invasive surgery [34, 35]. The dVRK is a telerobotic surgical research platform that allows 3D visualisation, and position, velocity and current control. The dVRK has a Master Tool Manipulator (MTM), which is controlled by the operator and used as input for the system while a stereo viewer provides visual feedback to the operator.

### Control and communication

**Fig. 3:**
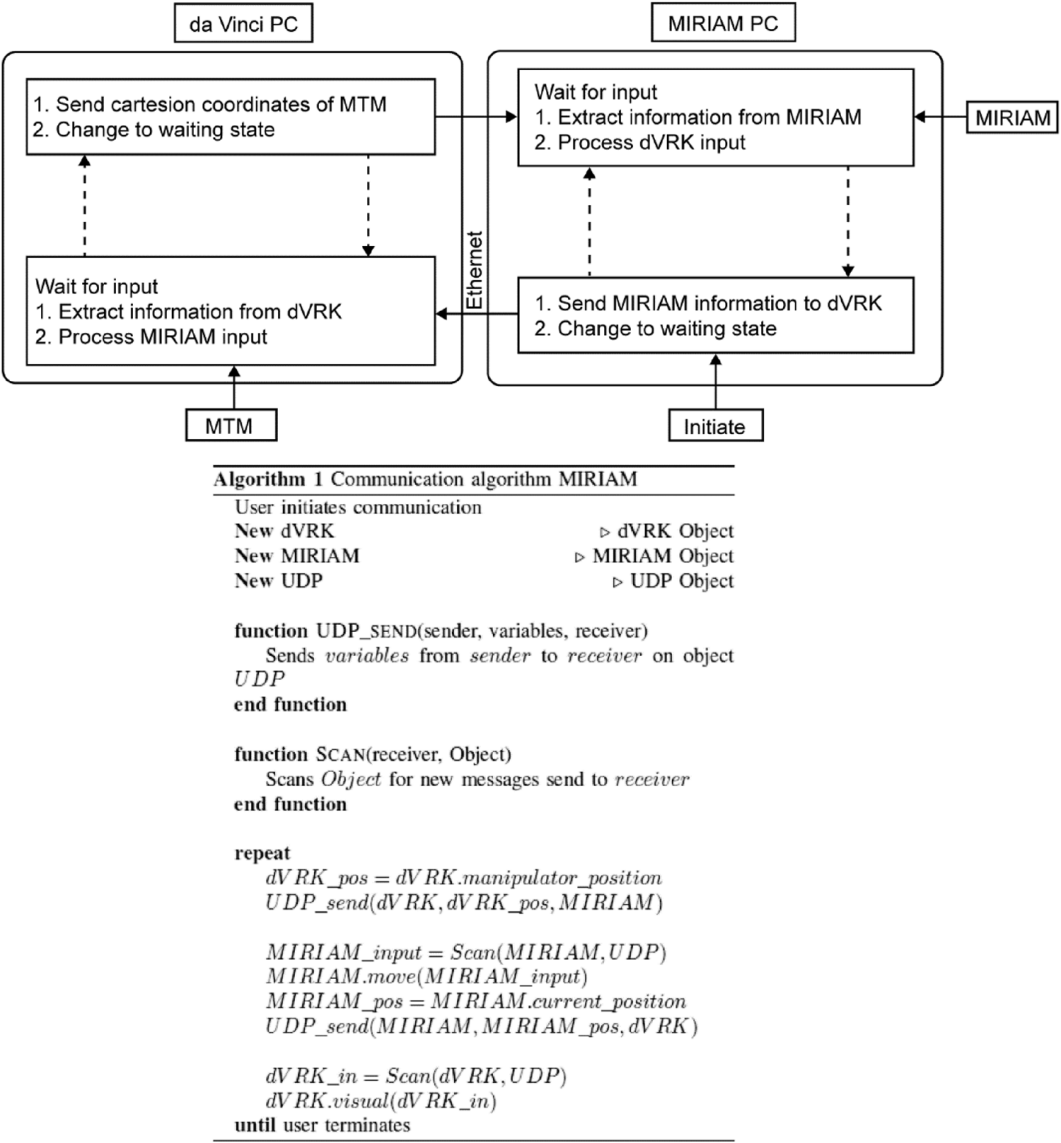
Communication protocol between the MIRIAM system and the dVRK. The system is initiated by the operator on the MIRIAM PC, information about MIRIAM is sent to the daVinci PC and the MIRIAM PC returns to a waiting phase. The daVinci PC processes the input, sends information about the MTM of the dVRK to the MIRIAM PC and returns to the waiting phase. The MIRIAM PC processes the information and the loop repeats itself. As both systems wait for an input from the other PC they will remain in synchronization. Information is exchanged between the two PCs via Ethernet. The dashed arrows relate to the first steps, the other arrows relate to the second steps

A continuous exchange of information between the MIRIAM system and the dVRK allowed for actuation of the steerable needle. The Z direction controlled the needle insertion, whereas the X direction controlled the degree of steering (i.e., the XZ steering plane). Velocity controlled steering was implemented using the equation:

1 $${\varvec{v}} = k \cdot \Delta {\varvec{x}}{,}$$

where ${\varvec{v}}$ is the velocity (m/s) of the needle base, $k$ is a scaling constant, and $\Delta {\varvec{x}}$ is the change in MTM position in the X direction (m). The continuous exchange of information through the local area network (LAN) provides plug and play operation of the system, while the user datagram protocol (UDP) ensures fast communication. The dVRK side uses the open-source Robotic Operating Software (ROS) package version Noetic Ninjemys and the MIRIAM side uses MATLAB 2016 with a protocol handling the communication between the MIRIAM system and the dVRK (Fig. 3). Continuous visual feedback collected from the MR scanner is provided to the operator. The actual position and the next position of the MIRIAM system, proposed by the operator using the MTM, are visualised and live updates of the movement are provided to the operator located outside the MR scanner room.

The software of the MIRIAM system is reprogrammed to switch between the steering and the insertion state triggered by the position of the MTM. Reorientation of the needle tip is performed in the steering state, while needle insertion is performed in the insertion state following the orientation of the tip. During operation, continuous path length estimations ensure that the carbon fibre-reinforced rods of the MIRIAM system have the length required to reach the pre-defined target. This path length estimation is evaluated for every 50 cycles between the MIRIAM system and the dVRK system to provide fast and accurate communication. In the steering state, the position from the MTM is continuously received and converted to the deviation of the MTM from the starting position ($\Delta {\varvec{x}})$. The velocity value is converted into a discrete function via:

2 $${\varvec{v}} = \frac{{{\varvec{pos}}_{{\varvec{k}}} - {\varvec{pos}}_{{{\varvec{k}} - 1}} }}{h} ,$$

For every time step ($h$) with a frequency of 100 Hz, Eq. (3) can be applied by combining Eqs. (1) and (2):

3 $$\user2{pos}_{\user2{k}} = \user2{pos}_{{\user2{k - 1}}} , + 0.01 \cdot k \cdot \Delta \user2{x},$$

where $k$ is a scaling constant and $\Delta {\varvec{x}}$ is the change in position of the MTM in the X-direction related to the starting position. The scaling constant is experimentally derived ($k=0.005$) to provide control stability after evaluating the end effector movement with various constants (1, 0.1, 0.01 and 0.005) Eq. (2) is used to change the position for the MIRIAM system based on the input of the MTM. MIRIAM X and Z coordinates were coupled so that the needle base always moved in an arc-like manner around the needle guide. This prevented needle insertions or retractions (Z coordinate) when steering conditions were changed (X coordinate). For insertion, $\Delta {\varvec{x}}$ in Eq. (3) is replaced by $\Delta {\varvec{z}}$. This method directly translated forward motion of the MTM to the insertion of the steerable needle, while backward motion of the MTM caused needle retraction.

### Experimental set up

The MIRIAM system with the steerable needle attached was placed in the MR scanner while the dVRK for the control was at a remote location. The components of the system and a schematic of the targeting experiment in a prostate phantom are shown in Fig. 2.

### Prostate phantom

The prostate phantom contained targets ventrally located to the urethra at a depth of 80 to 87 mm. The prostate phantom is based on an anonymous patient dataset containing a 65.8 cc prostate and is fabricated of 10 wt.% porcine gelatine (Dr. Oetker, Bielefeld, Germany) approximating the Young’s modulus of prostate tissue (58.8 ± 8.2 kPa) [36]. The MR images are segmented in SolidWorks (Dassault Systèmes SOLIDWORKS Corp.) and a mould is 3D-printed for manufacturing the prostate gland. The phantom contained a prostate gland, adipose tissue, pubic arch of acrylonitrile butadiene styrene, urethra (ø 5 mm silicone rubber rod), rectum and four ø 1 mm targets (carbon fibre-reinforced tubes). To distinguish between the prostate and the adipose tissue 5 g of contrast powder (Sudan orange G, Carl Roth, Karlsruhe, Germany) is added to the prostate.

### Experimental protocol

**Fig. 4:**
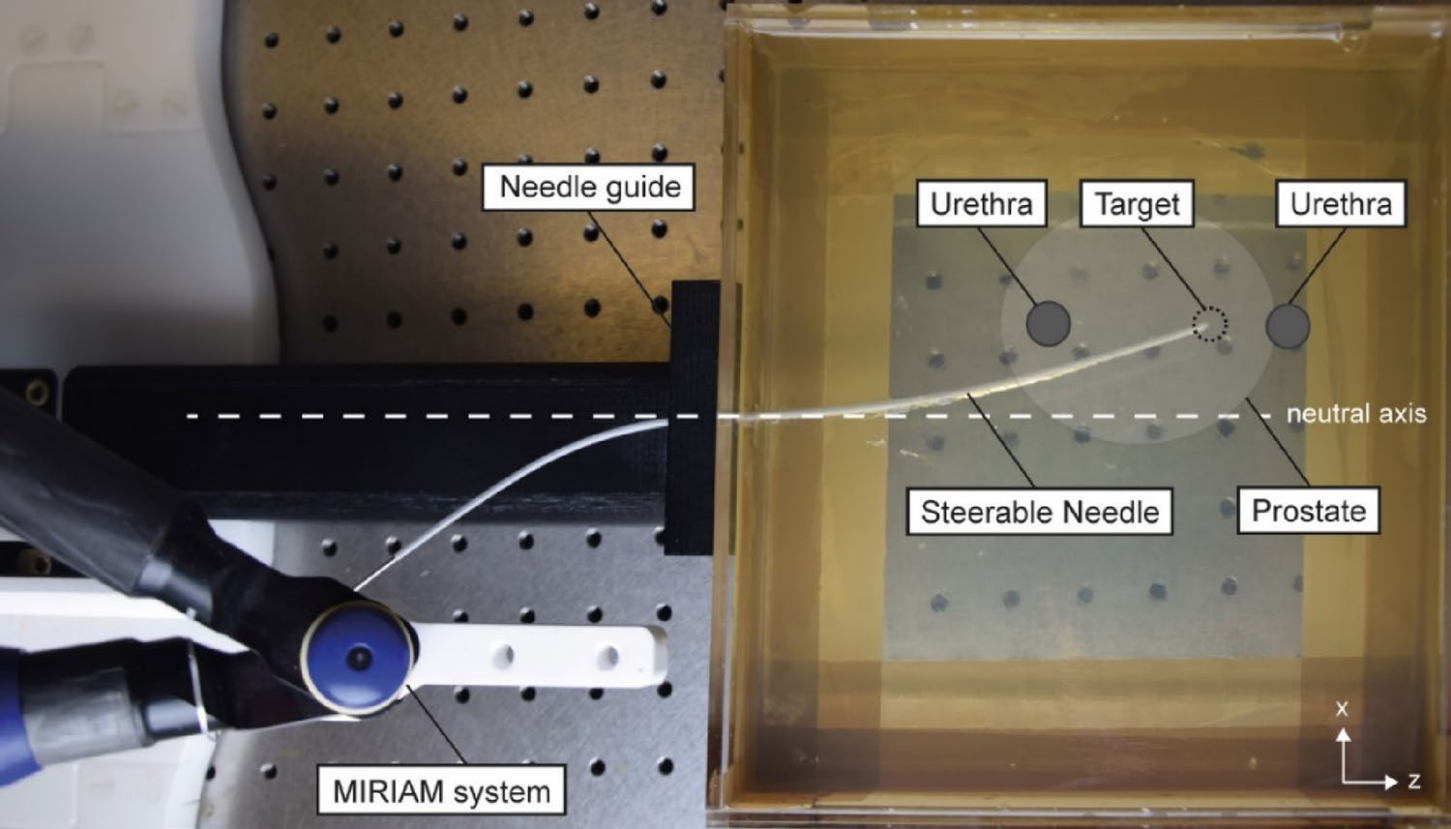
Steering example with the steerable needle attached to the MIRIAM system. The steerable needle is inserted in a medium along a curved trajectory to reach a pre-defined target while circumventing the intermediate structure. Steering is applied after penetrating the medium to obtain steering from the neutral axis

The ability to steer the needle along a curved trajectory while avoiding intermediate structures and reach the pre-defined target with the teleoperated system is evaluated for ten insertions in a prostate phantom under MR-guidance (MAGNETOM Aera 1.5 T, Siemens Healthineers, Erlangen, Germany). The curvature of the trajectory towards the pre-defined target remained with the operator and adaptations of the input position for the MIRIAM system were allowed during insertion to accurately reach the target. Figure 4 shows an example of steering with the steerable needle attached to the MIRIAM system.

### Data acquisition and analysis

**Fig. 5:**
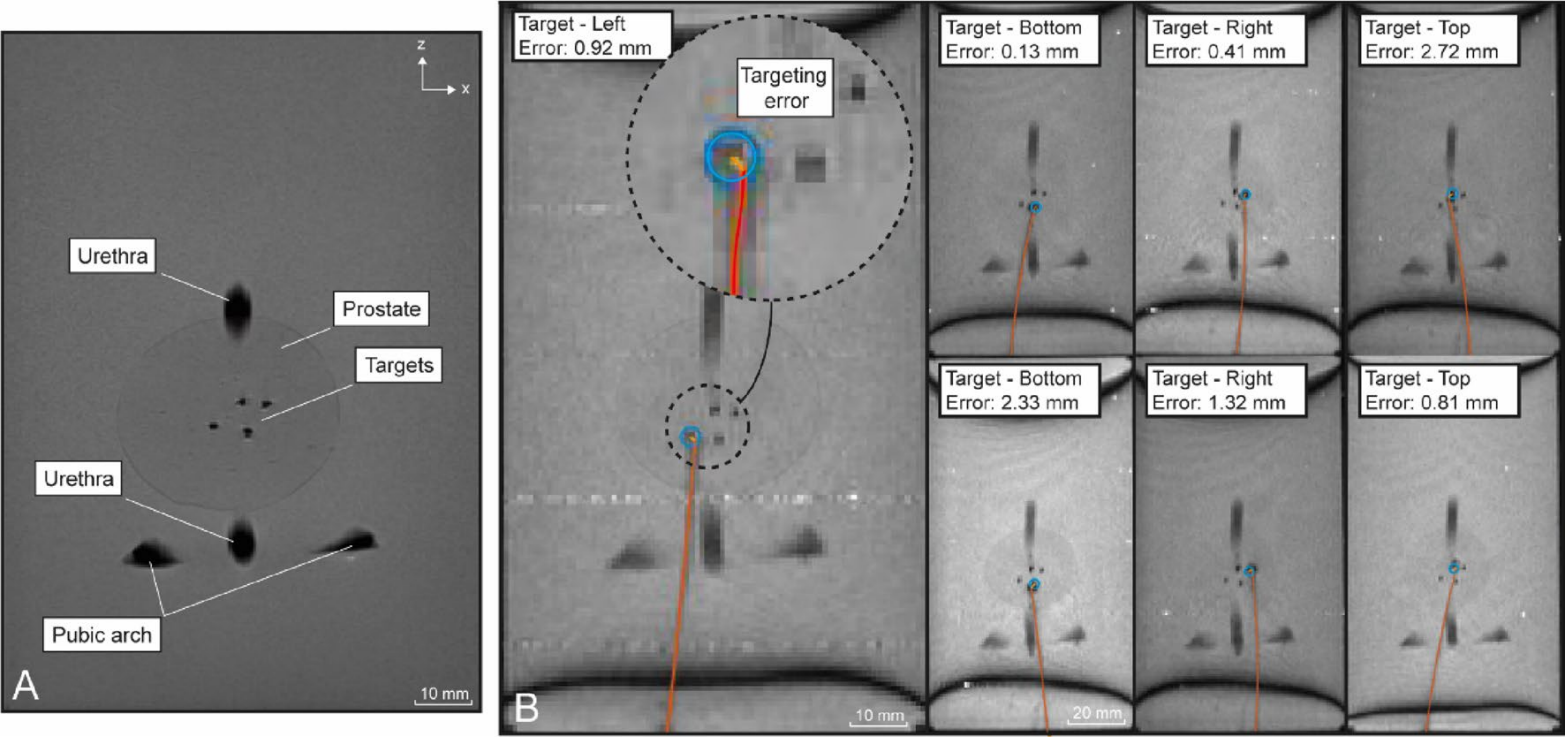
MR scans in coronal plane of the inserted steerable needle in the prostate phantom. The opacity of the prostate is changed for visualisation purposes. **A** High resolution MR scan, **B** Real-time MR scans indicating the segmented curved trajectories of the steerable needle (red), the targets (blue) and the targeting errors (orange) for several insertions. See supporting information – Video 2

Real-time T1-Weighted TurboFlash in 2D (T1W-TF2D) scans were acquired. The imaging parameters were: FOV = 191 × 272 mm; flip angle = 70°; TR/TE = 250.85/1.24 ms; voxel size = 1.31 × 1.31 mm; slice thickness = 8 mm and number of slices = 1. High resolution scans were acquired with imaging parameters: FOV = 150 × 180 mm; flip angle = 160°; TR/TE = 5590/101 ms; voxel size = 0.56 × 0.56 mm; slice thickness = 3 mm and number of slices = 19. The relatively low resolution of the real-time scans challenged evaluation of the needle tip position. Thus, a second order polynomial fit was made to the simple path curvature to assess the targeting error (see Fig. 5). The error is assessed in-plane in 2D by the Euclidean distance between the target and the determined end-position of the steerable needle tip on the coronal slice.

## Results

The communication between the MIRIAM system and the dVRK system, and the control of the steerable needle by the integrated system were successful as teleoperated adaptive steering could be performed with dVRK in the MR environment (see supporting information – Video 1 and Video 2). Figure 5A shows a high resolution MR scan of the phantom in coronal plane and Fig. 5B shows real-time MR scans with segmented needle trajectories and the resulting 2D-error between the needle tip and target positions.


Supplementary file1 (MOV 87782 KB)Supplementary file2 (MOV 2714 KB)

In the steering state, the maximum velocity in X-direction of the needle base was ~ 1 mm/s, while the maximum insertion velocity was ~ 20 mm/s. The latency for control between the two systems was on average 8.5 ms (range: 0.43–51.7 ms) and MR scans were sent to the operator every 2 s. The pre-defined target was reached for all ten insertion after 60–180 s without any reinsertion required and following different curved trajectories with an average targeting error of 1.2 ± 1.0 mm (Table 1).

**Table 1:** Absolute targeting errors in 2D of the steerable needle insertions in the prostate phantom

Trial	Target	Targeting error (mm)
1	Left	0.92
2	Left	1.72
3	Bottom	0.13
4	Bottom	2.33
5	Right	0.41
6	Right	1.32
7	Right	0.56
8	Top	2.72
9	Top	0.81
10	Top	1.12
Mean ± σ		1.2 ± 1.0

σ = standard deviation

## Discussion

This work presented teleoperated needle steering in an MR environment for the purpose of HDR prostate BT. We showed the feasibility of the teleoperation system and high targeting accuracy of the steerable needle in a prostate phantom. The system integrated three subsystems: (1) the steerable needle, (2) the MIRIAM system and (3) the dVRK, while MR scans provided the ability to continuously monitor the steerable needle trajectory and adapt the level of steering if required during the procedure. The system is plug and play which allows it to be used in the MR scanner room with minimal adaptations to the set up. The device places standard ProGuide needles that are directly usable in the subsequent radiotherapeutic workflow. Only positioning of the MIRIAM system in the MR scanner and the HDMI connection for the MR signal are required.

The needle targeting accuracy we obtained in this work is comparable to other studies with MR-compatible BT robots [37]: needle positioning errors of 0.9–3.2 mm were reported of which some studies evaluated oblique needle insertions [21]. All errors remained below the limit of 3 to 4 mm used by Borghede et al*.* [38] when exceeded requiring needle reinsertion. More recently, the CoBra research group developed an MR-compatible robot suitable for LDR prostate BT with active steerable needles to bypass intermediate structures. The system incorporated a module with piezoelectric motors to be mounted on the robot for BT needle steering [39], while our system required attachment of the steerable needle only. As a result of increased accessibility of the target volume and the promising dosimetric outcomes in earlier studies [22–25], one can emphasize that our system ensures a sufficient dose coverage in the prostate.

The integrated system in this work can be classified as a Level II system as “a human specifies general moves or position changes and the machine decides specific movements of its actuators” [21]. To upgrade this human-in-the-loop system to a level IV system in which “the machine will create and complete all its tasks without human interaction”, a predictive model with path planning and closed-loop control should be integrated using visual information of the MR scans. Apart from this, optimisation of the teleoperation system is required. Firstly, the MIRIAM system has limited movement possibilities in the Y-direction while for clinical application steering in X- and Y-direction is required so that for example pubic arch interference can be overcome. Currently, we are working on scan plane control to automatically reposition the image plane, which will allow for future 3D steering studies. Secondly, the dVRK system allows for 3D visualisation and haptic feedback while not implemented in this work. As the needle has a non-negligible stiffness, the X–Z motion coupling, to move the needle base in an arc-like manner around the entry point in the needle guide, was only sufficient by approximation: slight axial displacements of the needle tip were still observed when the level of steering was adjusted. Thirdly, it was previously shown that in particular open or hollow nitinol structures can cause artefacts in MR images, caused by shielding effects [40, 41]. In our case, we did not see such effects, as our most inner component was made from nitinol and we used a polynomial fit on the real-time scans to assist in error determination. Nevertheless, it is recommended to approximate more complex needle trajectories using a higher order polynomial, and to determine the targeting error in 3D. Finally, we recognize that our validations were performed in a static and homogeneous phantom environment. True system validation will require a continuation of this work in a more realistic setting.

The ability to steer the distal tip of the steerable needle allows for counteracting perturbations and follow a pre-defined trajectory. With this, the teleoperation system can be suitable for other treatments such as brachytherapy of the cervix, and biopsies or focal laser ablation of liver and prostate cancers, with only minor changes to workflow and without the need to develop a completely new robotic system.

## Conclusions

This work shows the feasibility of teleoperated needle steering for BT interventions in a prostate phantom. The novelty comprises the system integration of an actively steered needle, the MIRIAM system, and the da Vinci Research Kit (dVRK), in combination with MR-guidance. The mode of operation of the system was validated and a high targeting accuracy was demonstrated in a prostate phantom. MR scans provided the ability to continuously visualise both the steerable needle and the target position. The teleoperated system allowed for adaptive steering of the needle thus compensating for deviations from the pre-defined trajectory, avoiding intermediate structures and reaching previously inaccessible target locations.

## Data Availability

All data supporting the findings of this study are available within the paper and its Supplementary Information.
